# Role of IABP in OPCAB - a vettath’s modification

**DOI:** 10.1186/1749-8090-8-S1-O207

**Published:** 2013-09-11

**Authors:** M Vettath, EI Tazhakuni, AK Veluchamy

**Affiliations:** 1 Dept. of Cardiothoracic & vascular surgery, MIMS, Kerala, India

## Background

In off-pump coronary artery bypass (OPCAB) surgery, the most critical complication is hemodynamic deterioration, which can occur during displacement of the heart to expose the target vessels. Pre-operative intra-aortic balloon pump (IABP) therapy has shown to improve cardiac performance and facilitates access to the target coronary artery while maintaining hemodynamic stability, especially in high-risk patients. But the need for using IABP in the so called high risk patients has been questioned in our group of patients. We have therefore modified the role of IABP in our OPCAB patients, thereby avoiding the conversion to heart lung machine.

## Methods

We have performed more than 3000 OPCABs in our center, over the last ten years, by a single surgeon (MPV).We had used 173 IABPs in the last ten years, and only one patient did go to the ICU with IABP in the last 5 years and over 2000 patients. All of them were removed from the operation theater itself.

## Results

We have been able to modify the role of IABP in OPCAB in our setting. We had inserted IABP initially in all the high risk group of patients as described in literature. But what we noticed was that patients with low ejection fraction and patients with critical left main coronary artery disease were not the ones who needed to have IABP support. It was the patients who had ongoing ischemia, with good left ventricular function, and patients with ST depression pre- operatively; who needed IABP support to perform complete revascularization of all its territories.

## Conclusions

The IABP appeared to facilitate the intraoperative hemodynamics in our series of patients. This was evidenced by improved hemodynamic stability and virtual elimination of the need for inotropic support during the dislocations of the heart needed for exposure and construction of distal anastomoses. What we have modified here is to introduce the IABP, as soon as any hemodynamic compromise is envisaged. This allows us to perform OPCAB in virtually all coronaries around the heart, maintaining perfect hemodynamics. And once the distal anastomosis is performed, the IABP is put on a standby mode to perform the proximal anastomosis. We have been able to then remove the IABP in the operation theater (OT) itself. This technique has enabled us to avoid conversion to the heart lung machine.

